# Synchronous online focus groups in health research: application and further development of methodology based on experiences from two mixed-methods research projects

**DOI:** 10.1186/s13104-023-06288-0

**Published:** 2023-02-20

**Authors:** Jonas Lander, Hala Altawil, Elise-Marie Dilger, Anna Levke Bruett, Lara Marleen Fricke, Dyon Hoekstra, Cosima John, Christian Krauth, Kathrin Krüger, Kristina Schaubert, Julia von Sommoggy, Annett Thiele, Marie-Luise Dierks

**Affiliations:** 1 Institute of Epidemiology, Social Medicine and Health Systems Research, Hannover Medical School, Hannover, Germany; 2 Department for Health Services Research, University of Oldenburg, Oldenburg, Germany; 3 Institute for Special and Rehabilitation Education, University of Oldenburg, Oldenburg, Germany; 4 Institute for Epidemiology and Preventive Medicine, University of Regensburg, Regensburg, Germany

**Keywords:** Online focus groups, Digital focus groups, Synchronous online focus groups, Focus group methods; health research

## Abstract

**Objectives:**

Focus groups used for data collection in health research are increasingly conducted online. In two multi-center health research projects, we applied available methodological instructions for synchronous online focus groups (SOFGs). We describe necessary changes and specifications regarding the planning (recruitment, technology, ethics, appointments) and conduct (group composition, moderation, interaction, didactics) to enhance knowledge about the planning and conduct of SOFGs.

**Results:**

Recruiting online proved to be challenging and necessitated direct and analogue recruiting, too. To ensure participation, less digital and more individual formats may be offered, e.g. telephone calls. Explaining verbally the specifics of data protection and anonymity in an online setting can foster participants’ confidence to actively engage in the discussion. Two moderators, one moderating, one supporting technically, are advisable in SOFGs, however, due to limited nonverbal communication, roles and tasks need to be defined beforehand. Participant interaction is central to focus groups in general, but sometimes difficult to achieve online. Hence, smaller group size, sharing of personal information and moderators increased attention to individual reactions appeared helpful. Lastly, digital tools such as surveys and breakout rooms should be used with caution, as they easily inhibit interaction.

**Supplementary Information:**

The online version contains supplementary material available at 10.1186/s13104-023-06288-0.

## Introduction

Digital tools to plan and conduct research may offer many advantages, not only in times of the COVID-19 pandemic [1]. In qualitative health research, focus groups (FGs) are an established methodology, not least as the interplay among participants can result in a more comprehensive understanding than simply gathering individual statements [2–4]. Several publications report on the application of online focus groups (OFGs) in health research [5–9], and there is emerging evidence about applied OFG methods [10]. One main difference between these studies is the format used, which is either textual (e.g. chats on closed social media platforms) or audiovisual (e.g. Zoom meetings), and asynchronous (e.g. an online message board) or synchronous (e.g. a live meeting). Recent studies state that asynchronous text-based OFGs have been to date the most-used format [2, 11], because of the ease of participation. Synchronous audiovisual OFGs (SOFGs), which are the focus of this work, resemble the original in-person format more closely, but are also more challenging [12, 13]. We expect many researchers to be interested in the benefits of SOFGs, particularly in terms of organization, time, economic resources, and overcoming physical distance, even post-pandemic. However, only a few academic contributions address the overarching issues of planning and conducting SOFGs. Further, although methodological instructions exist (e.g., [2, 13, 14]), there is little empirical evidence on whether these are applied, and if so, to what extent they are appropriate for SOFGs in health research. Hence, based on our own experience, we aim to describe how far existing instructions are suited to the planning and conduct of SOFGs, and to suggest modifications where necessary.

## Main text

### Methods

From October 2020 to September 2021, we conducted a total of n = 37 SOFGs across two health research projects. One was related to the health information behavior of parents of infants (project A [15]), the other to the experiences and needs of people with multiple sclerosis and health professionals in the healthcare system (project B [16]) – the contents are presented in additional file 1. We enrolled a total number of n = 125 participants across both SOFG projects (respectively n = 77 and n = 48). As the initial plan was to conduct FGs in person at each project site, we devoted particular attention and effort to the conversion to SOFG format. During technical planning, we selected a suitable video-conferencing platform (that offered e.g., private chat, hand raising, document sharing, and a virtual whiteboard), agreed data protection aspects with a data safety expert, modified the initial informed consent form, and developed tutorials (project A = video- and text-based, project B = text-based). To ensure smooth running, we conducted pre-tests and offered pre-meetings to explain the platform and clarify any questions on the use. Based on the results of the pre-test and the available methodological instructions, we made various adjustments to the traditional FG format, outlined in more detail below.

### Results

The following sections describe the main steps in our planning (recruitment, ethics, technology, appointments) and conduct (duration, moderation, interaction, didactics) of the SOFGs. In each, we briefly summarise recommendations from available SOFG instructions, and explain how we implemented them, with any modifications we found useful. Table 1 provides an overview of which topics are already covered by existing methodological instructions and indicates in how far we had to adapt respective instructions and suggestions.

**Table 1:** Summary of SOFG challenges, recommendations, and adjustments

	Topic	Specific challenge	Recommendations in literature?	Recommendation applied?	Recommendation changed or made more detailed?
1	Recruiting	How to reach people who cannot or do not want to participate online?	Partly - Embrace diverse options of online recruitment [18]	Partly	Yes - direct, analogue recruitment (particularly for hard-to-reach-groups)
2	Ethics	How to ensure participants do not worry about anonymity, privacy, confidentiality?	Yes - Adjust, specify informed consent document [2]	Yes	Yes - outline privacy aspects verbally at the beginning of a SOFG
3	Technology	How to handle participants who are less technically literate or not used to an online conversation? How to ensure technical functioning?	Yes - pre-test [21] - extra time [2] - technical assistant [21] - chat [18]	Partly	Yes - offer analogue (non-digital) conversation alternative
4	Appointments and time	Which time slots suit participants best and how can “no-shows” be avoided?	Yes - online scheduling [18] - offer several timeslots [18] - over-recruit [12] - reminders [21]	Partly	Yes - prior in-person conversation to agree on appointment, or - invite more participants than needed to one SOFG appointment (over-invite)
5	Duration	How long should a SOFG last?	Partly - Communicate it clearly [21]	Yes	Yes − 60–75 min limit, longer meetings are possible but require participants’ agreement
6	Moderation	How can moderators communicate among each other?	Yes - Basic participation rules [13]	Partly	Partly - assign moderation parts and topics in advance - subdivide moderation roles (e.g. technical moderator; answering of questions) - use of a separate chat
7	Interaction	How to enable effective interaction online?	Yes - Introduce exercise [2] - Devise subgroups [13] - introduce interaction [20] - Digital tools [2]	Partly	Yes - consider group composition and participant characteristics as central elements for interaction - maintain interaction throughout moderation
8	Didactic methods	Which methods work well in a SOFG setting?	No		- avoidance of unfamiliar digital tools - use of (only) self-evident tools - focus on participant discussion/interaction instead of introduction of additional methods

#### Recruitment

Methodological instructions suggest that, because of the sheer number of options and channels for recruitment, recruiting participants online offers the ability to reach broader and more diverse populations [13, 17, 18]. However, during our own recruitment efforts (using e.g. Facebook, Instagram, self-help organisations), we found contacting hard-to-reach groups particularly difficult: invitations to participate in the study were easily overlooked or disregarded by people without a specific interest in health research. It was therefore necessary (and effective) to add recruitment channels that allow more direct contact with and more detailed explanation to potential study participants, e.g. via patient representation associations or social workers in childcare institutions who could approach potential study participants in person.

#### Ethics

The available literature points out that ethical aspects of OFGs are not fundamentally different from those of in-person FGs [19]. Nevertheless, aspects need to be specified in the consent forms according to the format, e.g. the meaning of anonymity or pseudonymity in an online audiovisual session [13, 19, 20]. In our studies, we received hardly any prior questions or feedback from participants regarding these matters, and were therefore uncertain about whether participants deem these issues relevant in an online environment. Hence, and because of the potentially sensitive subjects of discussion, and other issues such as other family members being recorded if they are in the same room, it seems vital to address at least the most important issues verbally at the start of a meeting, or even during pre-meeting contacts with participants. This explicit attention may also help participants to feel confident about openly contributing to the group discussion.

#### Technology

The main concern regarding technology in SOFGs is that participants who are unable to participate in research conducted online will be excluded from the respective research projects or that unexpected technical difficulties arise during participation, resulting either in sampling limitations or missing out on important participant contributions, respectively. The available literature identifies various ways to overcome technical difficulties, notably the use of prior instructions and/or testing, allowing extra time at the beginning of a SOFG to ‘technically set up’ each participant, and the provision of a technical assistant for questions during the meeting [2, 18, 21]. It proved helpful to not only rely on digital methods, but to apply e.g. individual telephone interviews, too, as this was effective to persuade those to participate who – specifically – lacked the technical means or felt unprepared for online participation.

#### Appointments

In an online research setting, the ‘threshold’ for not showing up or cancelling a meeting spontaneously seems lower – for a variety of individual reasons which were not enquired in more detail during our own research. Therefore, it is vital to think about how to avoid ‘no-shows’ and last-minute cancellations. While these can never be completely avoided, it has proven helpful not only to send reminders [21] but also to ask participants for a short confirmation by email and/or participation in a technical pre-test, which may increase the sense of commitment when a personal meeting has taken place a priori. A strategy we applied to achieve a good SOFG group size was to invite n = 7 participants to a specific appointment, for which in almost all cases only n = 3–5 participants will confirm. Any ‘missed’ participants can then be re-invited for upcoming appointments (project A). A different way of filling the sample was to contact each individual who expressed particular interest personally and agree on one specific appointment (project A and B).

#### Duration

During our own pre-testing, we found that in an online environment a ‘traditional’ two-hour meeting – as is common for in-person FGs – may be too long. Apart from advice to clearly communicate the length [21], there are few concrete suggestions as to the ideal length of an SOFG. We found that 60–75 min seems attractive, particularly for busy people such as parents of infants, or when the topic of discussion is more abstract. Longer meetings (e.g., 90 min) may be considered when participants are personally concerned with the topic, hence motivated to discuss in more detail, e.g., healthcare system barriers for chronically ill people (here: multiple sclerosis patients), but then require breaks from participating in a (comparatively more) physically and mentally demanding online setting, particularly for those with current health problems.

#### Moderation

The available literature often emphasizes the role of moderators in guiding and leading the discussion [2]. Another challenge we experienced was communication among moderators of a jointly-moderated SOFG (e.g., main and technical moderator), as they are just as hampered as the participants by the lack of direct contact and visual cues, resulting in difficulties in spontaneous and immediate coordination. In addition to suggestions such as the introduction of basic group rules or strategies to handle off-topic or overly active contributors [13], we recommend that each discussion theme – or, alternatively, each moderation task – be assigned to a single moderator, and to communicate this to participants during introduction: e.g., moderator A introduces main themes, moderator B handles follow-up questions. Using a private (moderator) chat proved helpful, too. While small(er) groups could also be led by only one moderator, the important task of achieving and maintaining interaction in an online setting (see below) can be better achieved by having two moderators. This helps to uphold and enhance lively discussion, which tends to easily stagnate online.

#### Interaction

Participant interaction is critical for successful FGs, but in SOFGs it is often limited, due to factors such as missing nonverbal communication or the lack of quick annotations and side talks [12]. Previous research often discusses aspects such as group size, group composition and moderation independently, but they should be considered together for their impact on interaction. Therefore, we propose: Firstly, ensure group participants share at least one specific characteristic (e.g., disease status, cultural background) to facilitate the feeling of togetherness within the group despite physical distance. Secondly, pay special attention to a small group size (e.g. n = 4–6, depending on aim and composition of SOFG), as interaction with each other becomes more difficult online with each additional member; and finally, moderators should actively solicit participants’ response to what is being said, to keep the discussion going.

#### Methods (didactics)

There are many didactic methods, from both traditional in-person FGs, e.g. participants brainstorm ideas with their seat neighbor, or from available OFG instructions, e.g., the use of digital collaboration tools. However, we found that, especially as SOFGs are still new to most participants, additional methods should be introduced with caution. First, they require additional time, making it harder to keep SOFGs brief (see above). Second, they easily distract from efforts to increase interaction, as participants need time to understand what they are supposed to do. Thirdly, even supposedly “easy” tasks like using a weblink can create technical difficulties, e.g. for mobile device users who need to switch among applications. Therefore, we suggest introducing only self-evident methods, e.g., reading a short case scenario in the chat.

### Discussion

Since SOFGs are quite similar to in-person FGs, one may assume that the transition to an online format will be fairly smooth: e.g. the intended participants remain the same, hence changes to the recruitment or conduct would not be required. We found on the contrary that various aspects from recruitment to didactic methods require adjustment. Recently published OFGs [22–24] contain little to no description of how the online format was developed or adapted. A related important aspect is the availability and use of methodological instructions. First, during our own planning we noticed that potentially very helpful methodological instructions such as [2, 13, 21] are rather difficult to find in both academic and non-academic search engines and databases. Obvious search phrases like “online focus group guideline” failed to give satisfying results. To identify these sources, we had to expand the scope of the search, e.g. manually search the reference lists of previously-found articles. Second, while these sources are clearly helpful, especially in identifying the most relevant themes for planning, such as technology, group composition, moderation, etc., we found that applying suggestions was often only partially feasible, either because important specifications such as the number of participants, coordination among moderators, or practical ways to increase interaction are missing or contradictory, or because one´s individual study objectives and context require different approaches. Those who want to conduct a SOFG will benefit most from a step-by-step checklist that offers definite suggestions for each planning step; e.g. how to approach hard-to-reach individuals or measures that strengthen group interaction.

## Limitations

The SOFGs were conducted during the pandemic, thus the willingness to participate in a study that was conducted online might have increased. Further investigation with differently vulnerable groups and for other research topics and questions is needed, also to enable generalization regarding (a) post pandemic times and (b) experiences for other topics and study populations.

## Electronic supplementary material

Below is the link to the electronic supplementary material.

Supplementary Material 1

## Data Availability

The qualitative interview data underlying the initial studies reported here can be accessed upon request from the first author. The literature underlying the argumentation of this article is freely accessible (see reference list).

## Declarations

### Additional file 1

Focus group guidelines (.docx)

## List of abbreviations

FG: Focus group
OFG: Online focus group
SOFG: Synchronous online focus group
