# Supplementary material for: Synchronous online focus groups in health research: application and further development of methodology based on experiences from two mixed-methods research projects
Source: BMC Res Notes. 2023 Feb 20;16:18. doi: 10.1186/s13104-023-06288-0 (PMC9940673; doi:10.1186/s13104-023-06288-0)
Supplement: Supplementary file 1 — Supplementary Material 1 [file 13104_2023_6288_MOESM1_ESM.docx]

**Project A - focus group guideline**

**Part 1) Topic awareness, knowledge, relevance**

During the introduction, you have already briefly reported to what extent the topic of allergies already concerns you. Apart from that, whether there are already allergies in your family:

How often do you deal with the topic of "allergies in children" in your everyday life?

- Is the topic rather "important" or rather "unimportant" for you? Why?
- Which topics do you deal with concretely, which questions do you have when you think about your own child?
- Are other health issues related to your child important(er) to you?
- Do you already do something to prevent allergies in your child?

Have you ever read up on allergies in children?

- If so, what was the reason?
- What recommendations have you already heard or read about?
  - Where did you get the information?
- How do you decide for yourself which advice, which recommendations you listen to or when you classify information as "good" or "not so good"?
  - Do you even think about whether information is "good" or "bad"?
  - Do you have certain "standards"? (Expert opinions?)

- Apart from the topic of allergies, how else do you obtain information regarding child health?

Thank you very much so far! We have prepared another short example:

Imagine you have read on www.allergieinformationsdienst.de that among children and adolescents almost every fourth person suffers from an allergy. What is your first thought?

- What would you do to find out if it is true?
- Have you heard of that website before?

**Part 2) Information behaviour, challenges**

The next part is about how you deal with health information about your child and how easy or difficult you find it. For this purpose, we will now post a short "scenario" in the chat window, which we will also read out again.

Scenario A - Parents with and without special health risks for the child

Imagine you read on a well-known site on the internet that excessive washing, cleaning, disinfecting and avoiding pets increases the risk of allergies in your child. However, you yourself are very careful about hygiene. Now you are thinking about whether you should change something.

Scenario B - Expectant and new parents

Imagine you are at the paediatrician's or midwife's office. You talk about nutrition and learn that it doesn't make sense to avoid certain foods in babies and toddlers to reduce allergy risks. After the conversation, you want to find out more about this and read in a parenting blog that many parents avoid e.g. cow's milk, egg, wheat, nuts (from the 6th month). Now you are not so sure what is right.

Scenario C - Experienced parents

Imagine your child has an allergy and you have an appointment with the paediatrician. You talk about allergy treatment and learn that allergy vaccination is possible from the age of 6. At home, you want to get more information. In a forum, parents report that the therapy takes too long, is expensive and in the end doesn't help. Now you are not so sure whether the doctor's information is correct and whether the treatment would be good for your child.

What would you do now if you wanted to look into this information further?

- Where and how would you look for more information?
- Which sources would you trust, which would you rather not?

If you think of the internet,

- Which sites would you find helpful?
- Would you also "google" this or "children and allergies" in general?
- Do you generally use social media such as facebook, twitter, intstagram for these topics?

What about friends, family?

- Do you talk about the topic?
- How do you deal with other opinions?

Thinking again about weighing information (as in the example).

- What do you do when there are different opinions?
- What do you do when there are unanswered questions?

**Part 3) Needs, future design**

Finally, we would like to hear from you what you think "good information" on the health of your children should look like. We have prepared an example for this as well, namely...

(Participants read through one of the following pages (weblink provided by the moderator) and then discuss the following questions)

- What do you find good and not good about the page?
- Is anything missing?
- Is the information helpful to you? Why?
- If you have your own questions about "K. & A.", which source would you prefer?
- Is there a certain information format you prefer, e.g. videos, newsletter, website, information directly from the doctor?
- What constitutes "good" information for you?
- Do you have examples from other areas of child health that you find good information?
- If there were a new offer on the topic "How can I prevent allergies in my child", what would you like to see?

**Project B – focus group guideline**

**For patients:**

**Part 1: assistive device provision process**

1. There are many steps before the assistive device is in your hands and fits well. What experiences have you had in this process?

- How did the need to get an assistive device arise?
- How did the idea of getting an assistive device come about?
- How was it determined which assistive device to get?
- What can you tell about the financing and/or about the approval process with the health insurance fund/other payers?
- How satisfied are you with the delivery of the assistive device?
- After receiving the assistive device, what happens next?
- Did it happen that you wanted to receive an assistive device but did not get it? If so, what was the reason?
- What was the reason for the failure of the provision process with the assistive device?

**Part 2: Impact of assistive devices**

2. What can you do well again with the assistive device?

- What social or community activities can you participate in well again with the assistive device?
- What is the impact of the assistive device on your daily life at work?

3. Who or what makes it difficult for you to carry out the activities or participate in the areas of your life that are important to you?

- What difficulties in the activities and participation in the areas of life that are important to you are perhaps also experienced because of the assistive device?

4. Who or what facilitates the activities and participation in the areas of life that are important to you?

5. Imagine you were talking to someone who does not have MS and does not use an assistive device.

- How would you describe to the person the importance of the assistive device to the quality of life and wellbeing of people with MS?

6- In contrast, in what areas of your life do you not use your assistive device?

- What is different about these situations compared to the situations where you use it?

**Part 3: Suggestions for improvement**

7. What do you think needs to change to improve the provision of assistive devices?

8. Do you have any other important comments or additions regarding the provision of assistive devices for people with MS that we have not yet addressed in this focus group?

9. We have talked a lot today about the walker, the manual wheelchair and the electric wheelchair/scooter. Which assistive devices do you think should also be explored in more depth?

10. Which stakeholders of the provision process of assistive devices do you think we should invite to future focus groups?

The order of the questions was flexible depending on the course of the focus group discussion

**For healthcare professionals:**

**Part 1: assistive device provision process**

1. Thinking back to your experiences with your MS patients or clients with MS, how would you describe the assistive device provision process?

- Even before the concrete assistive device is selected, the idea of receiving an assistive device - a wheelchair, for example - must first arise. What triggers the assistive device provision process?
- How is it determined which is the appropriate mobility-related assistive device is used?
- What role does the financing of the assistive device play in providing a person with MS with a mobility-related device?
- If we now jump a little further to the end, after the decision for a specific assistive device has been made. How do people with MS receive the actual mobility-related assistive device?
- We now imagine: the assistive device has been delivered and some time has passed. What does the supply look like then? Who can people with MS turn to for questions that arise in the follow-up?
- What reasons could there be for a person not receiving an assistive device?
- How would you describe the transitions between inpatient stay (rehab/hospital) and outpatient care if the person received a mobility-related assistive device during the inpatient stay?
- What is the relationship between the provision of assistive devices and remedies?
- If you compare the assistive device provision process of people with MS with other patient groups, what similarities and differences do you see?
- To sum up: how would you as a group of experts evaluate the provision process of assistive devices for people with MS?

**Part 2: Suggestions for improvement**

2. What do you think needs to change to improve the provision process of assistive devices for people with MS?

3. Do you have any other important comments or additions regarding the provision process of assistive devices for people with MS that we have not yet addressed in this focus group?

4. We have talked a lot today about the walker, the manual wheelchair and the electric wheelchair/scooter. Which assistive devices do you think should also be explored in more depth?

The order of the questions was flexible depending on the course of the focus group discussion.
